# Lack of Cytosolic Carboxypeptidase 1 Leads to Subfertility due to the Reduced Number of Antral Follicles in *pcd^3J-/-^* Females

**DOI:** 10.1371/journal.pone.0139557

**Published:** 2015-10-09

**Authors:** Ning Song, Nameun Kim, Rui Xiao, Hojun Choi, Hyo-Im Chun, Min-Hee Kang, Jin-Hoi Kim, Kunho Seo, Nagasundarapandian Soundrarajan, Jeong-Tae Do, Hyuk Song, Zhao-Jia Ge, Chankyu Park

**Affiliations:** 1 Department of Animal Biotechnology, Konkuk University, Seoul, South Korea; 2 Colleges of Veterinary Medicine, Konkuk University, Seoul, South Korea; 3 College of Animal Science and Veterinary Medicine, Qingdao Agricultural University, Qingdao, P.R. China; Ottawa Hospital Research Institute and University of Ottawa, CANADA

## Abstract

Females homozygous for the Purkinje cell degeneration mutation (*pcd*) are fertile, although the success rate is much lower than in the wild type. We performed detailed analysis of reproductive abnormalities of *pcd* females. The number of oocytes produced following exogenous gonadotropin treatment was much lower in *pcd* ^*3J-/-*^ females than in *pcd* ^*3J+/+*^ females. Furthermore, the estrous cyclicity of *pcd* ^*3J-/-*^ females according to the appearance of the vagina was almost undetectable comparing to that of the wild type. Histological analyses and follicle counting of 4- and 8-week-old *pcd* ^*3J-/-*^ ovaries showed an increase in the number of secondary follicles and a decrease in the number of antral follicles, indicating that AGTPBP1/ CCP1 plays an important role in the development of secondary follicles into antral follicles. Consistent with a previous analysis of the *pcd* cerebellum, *pcd* ^*3J-/-*^ ovaries also showed a clear increase in the level of polyglutamylation. Gene expression analysis showed that both oocytes and cumulus cells express *CCP1*. However, *Ccp4* and *CCP6*, which can compensate the function of CCP1, were not expressed in mouse ovaries. Failure of microtubule deglutamylation did not affect the structure and function of the meiotic spindle in properly aligning chromosomes in the center of the nucleus during meiosis in *pcd* ^*3J-/-*^ females. We also showed that the pituitary-derived growth and reproduction-related endocrine system functions normally in *pcd* ^*3J-/-*^ mice. The results of this study provide insight into additional functions of CCP1, which cannot be fully explained by the side chain deglutamylation of microtubules alone.

## Introduction

Purkinje cell degeneration (*pcd*) mutation is an autosomal recessive mouse mutation[1]. The *pcd* mice display multiple defects, including degeneration of cerebellar Purkinje cells [1, 2], retinal photoreceptor cells [3, 4], mitral cells of the olfactory bulb [5], and certain thalamic neurons [6, 7]. Moreover, the males show abnormalities in spermatogenesis and are thus sterile [1, 8]. It has been reported that the phenotypes of *pcd* mice are caused by mutations of the *Nna1* or *Agtpbp1* gene, but the cellular functions of the gene remain unclear [9]. It has been suggested that Nna1 functions as a carboxypeptidase, and thus *CCP1* was assigned as an alternate name for the gene [10]. Until now, 19 alleles of *CCP1* derived from spontaneous and induced mutations have been identified (S1 Table, http://www.informatics.jax.org). Several studies have shown that CCP1 functions as a tubulin deglutamylase [11–14], although it may also have other unknown functions. Berezniuk et al (2013) suggested that the altered peptide levels in adult *pcd* ^*3J-/-*^ mice could have resulted from altered proteasome function as a secondary effect of *CCP1* mutation [15]. A recent report showed that CCP1 processes not only glutamates but also C-terminal aspartates, suggesting that CCP1 might regulate protein-protein and protein-DNA interactions [16].

Because correct localization of cellular proteins and organelles is essential for proper cell functioning and their transport is tubulin-dependent, changes in tubulin processing could potentially explain the majority of defects found in *pcd* mice, including abnormal accumulation of polysomes [2], altered transcription and DNA repair [17, 18], endoplasmic reticulum stress [19], formation of axonal spheroids [20], mitochondrial dysfunction [21], elevated autophagy [22] and abnormal dendritic development [23]. However, other animal models with impaired tubulin polyglutamylation, such as ROSA22 mice, result in mislocalization of a molecular motor, KIF1A, and abnormality in tubulin-dependent trafficking and synaptic transmission [24–26], showing both similarities and differences with the phenotypes of *pcd* ^*3J-/-*^ mice. With the exception of tubulins, no other target genes of CCP1 have been demonstrated to date.

Although a number of studies have investigated the mechanisms underlying the phenotypes of *pcd* ^*3J-/-*^ mice and the functions of CCP1, several questions remain. It has been reported that *pcd* ^*3J-/-*^ adult females are fertile, but that they have difficulties in rearing the few litters they produce [1]. However, no detailed analyses of the reproductive abnormalities of *pcd* ^*3J-/-*^ females have been conducted. In this study, we performed analyses on *pcd* ^*3J-/-*^ ovaries, revealing that *pcd* ^*3J-/-*^ females show poor secondary to antral follicle development, and studied the possible cellular mechanism underlying the defects. Our study demonstrates the functional roles of CCP1 in female reproduction and suggests that other cellular defects, in addition to abnormality of tubulin depolyglutamylation, may be involved in *pcd* mutant mice.

## Materials and Methods

### Animal ethics

All animal experiments were approved and performed under the guidelines of the Konkuk University Animal Care and Experimentation Community [IACUC approval number: KU14081]. *Pcd* ^*3J*^ heterozygote (*pcd* ^*3J+/-*^) mice were purchased from the Jackson Laboratory (USA) and maintained under standard conditions (12-hour light/dark cycle), with food and water provided *ad libitum*. The animals used for these experiments were produced by crossing *pcd* ^3J^ heterozygotes. The genotypes and phenotypes of the animals were determined as previously described [27]. Animals were sacrificed in the CO_2_ chamber and tissues were harvested instantly.

### Superovulation

Eight-week-old females were stimulated with 5 IU pregnant mare serum gonadotropin (PMSG, Sigma-Aldrich, Missouri, USA) followed 48 h later by 5 IU human chorionic gonadotropin (hCG, Sigma-Aldrich, Missouri, USA) to induce superovulation of oocytes. The number of oocytes was counted 14–16 h after the second injection. The statistical significance was determined using Student’s *t*-test.

### Estrous cycle identification

To assess the possible abnormality of the estrus cycle in *pcd* females, visual observation of the vagina of 8 weeks old females was carried out according to the criteria previously described [28]. Briefly, in proestrus, the vaginal opening of mice is swollen, moist and pink. Moreover, the opening is wide and there are often wrinkles or striations along the dorsal and ventral edges. As the mouse enters estrus, the vaginal opening becomes less pink, less moist, less swollen and the striations are more pronounced. In metestrus a vaginal opening does not open wide, and is not swollen. Sometimes, white cellular debris may line the inner walls or partially fill the vagina. In diestrus, the vaginal has a small opening, very moist and closed with no tissue swelling.

### Western blotting

Ovaries from *pcd* ^*3J+/+*^ and *pcd* ^*3J-/-*^ mice were ground and homogenized in tissue lysis buffer [0.32 M sucrose, 0.01 M Tris (PH 7.4)] with protease cocktail inhibitor (Sigma-Aldrich, Missouri, USA) and 1 mM PMSF. Total proteins were extracted from the supernatant and quantified using a Bradford Assay [29]. Proteins were separated by SDS-PAGE and transferred to nitrocellulose membranes (GE Healthcare, Buckinghamshire, United Kingdom). Membranes were probed with a rabbit anti-polyglutamylation antibody (1:1000) [30] and a mouse anti-actin antibody (1:2000, Santa Cruz Biotechnology, Texas, USA), and blots were visualized using peroxidase-conjugated anti-rabbit and anti-mouse IgG antibodies (1:2000, Santa Cruz Biotechnology, Texas, USA), respectively.

### Reverse transcription polymerase chain reaction (RT-PCR)

Ovaries were punctured with a sterile blade and germinal vesicle (GV) oocytes were collected in M2 medium supplemented with 2.5 μM milrinone (Sigma-Aldrich, Missouri, USA) to maintain them at the germinal vesicle (GV) stage. After PMSG injection, we used a syringe to penetrate the ovary to obtain granulosa cells. Granulosa cells were washed with M2 medium and transferred to PCR tubes. Total RNA from the mouse ovaries were prepared using a miRNeasy Mini Kit (Qiagen, Hilden, Germany). Briefly, ovaries were ground in liquid nitrogen and homogenized in RLT buffer (Qiagen, Hilden, Germany) supplemented with 1% β-mercaptoethanol. The supernatant, mixed with 70% ethanol, was transferred into mini spin columns and total RNA was collected. For the isolation of total RNAs from GV oocytes and cumulus cells, ~100 GV oocytes and cumulus cells from three mice were prepared and the total RNA was isolated using a miRNeasy Micro Kit (Qiagen, Hilden, Germany), in the same manner as the miRNeasy Mini Kit. First-strand cDNA was synthesized with CycleScript RT PreMix (Bioneer, Seoul, Korea). *Gapdh* was used as an internal standard. The primers for the amplification of *CCP1* were described in Kim et al.[8] and *Ccp4* (also known as *Agbl1*) and *CCP6 (*also known as *Agbl4)* were described by Kalinina et al. [10].PCR products were run on 1.5% agarose gels and visualized using UV illumination.

### Quantitative Real-time RT-PCR

Quantitative PCR were carried out in a DNA Engine Opticon system (Bio-Rad, Hercules, CA, USA) using Rotor-Gene^®^ SYBR^®^ Green PCR kit (Qiagen, Venlo, Netherlands) in a 25 μl reaction volume (containing 12.5 μl 2 x Rotor-Gene SYBR Green PCR Master Mix, 1 μl each primer, 8.5 μl RNase-free water and 2 μl diluted cDNA of two-month-old ovary). The reaction conditions were an initial denaturation step of 95°C/30 s, followed by 40 cycles of 95°C/5 s and 60°C/30 s. All PCR reactions were carried out in biological and technical triplicate. A non-template control was also included in each run for each gene. The primers for the amplification of mouse *Amh* gene were: Forward primer CCACACCTCTCTCCACTGGTA; Reverse primer GGCACAAAGGTTCAGGGGG. *Gapdh* was used as an internal standard.

### Oocyte immunocytochemistry

Ovulated oocytes were collected from the oviducts of the mice 14–16 h following the hCG injection. For immunocytochemical analysis, the oocytes were briefly incubated with 0.3 mg/mL hyaluronidase in PBS and fixed for 30 min in 4% paraformaldehyde. The oocytes were then washed with washing buffer (PBS with 1% BSA) twice and treated with 0.5% Triton X-100 for 20 min. The oocytes were incubated with an anti-α-tubulin antibody (Santa Cruz Biotechnology, Texas, USA) for 2 h at room temperature. After washing twice, the oocytes were reacted with the Alexa Fluor ^**®**^ 568 goat anti-mouse IgG antibody (Life Technologies, California, USA) for 1 h at room temperature. The oocytes were washed three times and finally mounted with VECTASHIELD (Vector Labs, Burlingame, CA, USA) supplemented with DAPI.

### Histological evaluation and morphological classification of follicles

Ovaries used for histological analysis were collected from female mice at 4 and 8 weeks of age, respectively. Mice were treated with 5 IU PMSG and their ovaries were subsequently excised 9 h after the hCG injection to observe oocytes immediately before ovulation. Ovaries were removed, cleaned of extraneous tissue, and weighed. Tissues were then fixed overnight in 4% paraformaldehyde, dehydrated in ethanol, and embedded in paraffin. Sections were taken at a thickness of 6 μm, at intervals of 60 μm, and paraffin-embedded sections were mounted on slides. Hematoxylin and eosin (H&E) staining was performed for histological examination by light microscopy. Follicles were classified as previously described [31] into primordial follicles (an oocyte surrounded by a layer of squamous granulosa cells), primary follicles (oocyte surrounded by a single layer of cuboidal granulosa cells), secondary follicles (oocyte surrounded by two or more layers of granulosa cells with no antrum), or antral follicles (antrum within the granulosa cell layers enclosing the oocyte). Follicles were determined to be atretic if they displayed two or more of the following criteria within a single cross-section: more than two pyknotic nuclei, granulosa cells within the antral cavity, granulosa cells pulling away from the basement membrane, and/or uneven layers of granulosa cells.

### TUNEL analysis

Sections (6 μm) taken at intervals of 60 μm were prepared as described above and mounted on poly-L-lysine-coated glass slides, followed by deparaffinization and hydration. TUNEL reactions were performed using the In Situ Cell Death Detection Kit^®^ (Roche, Switzerland), according to the manufacturer’s protocol. Sections were counterstained with 4-6-diamidino-2-phenylindole (DAPI).

## Results

### Adult onset ovarian hypoplasia in *pcd* ^*3J-/-*^ females

**Fig 1:**
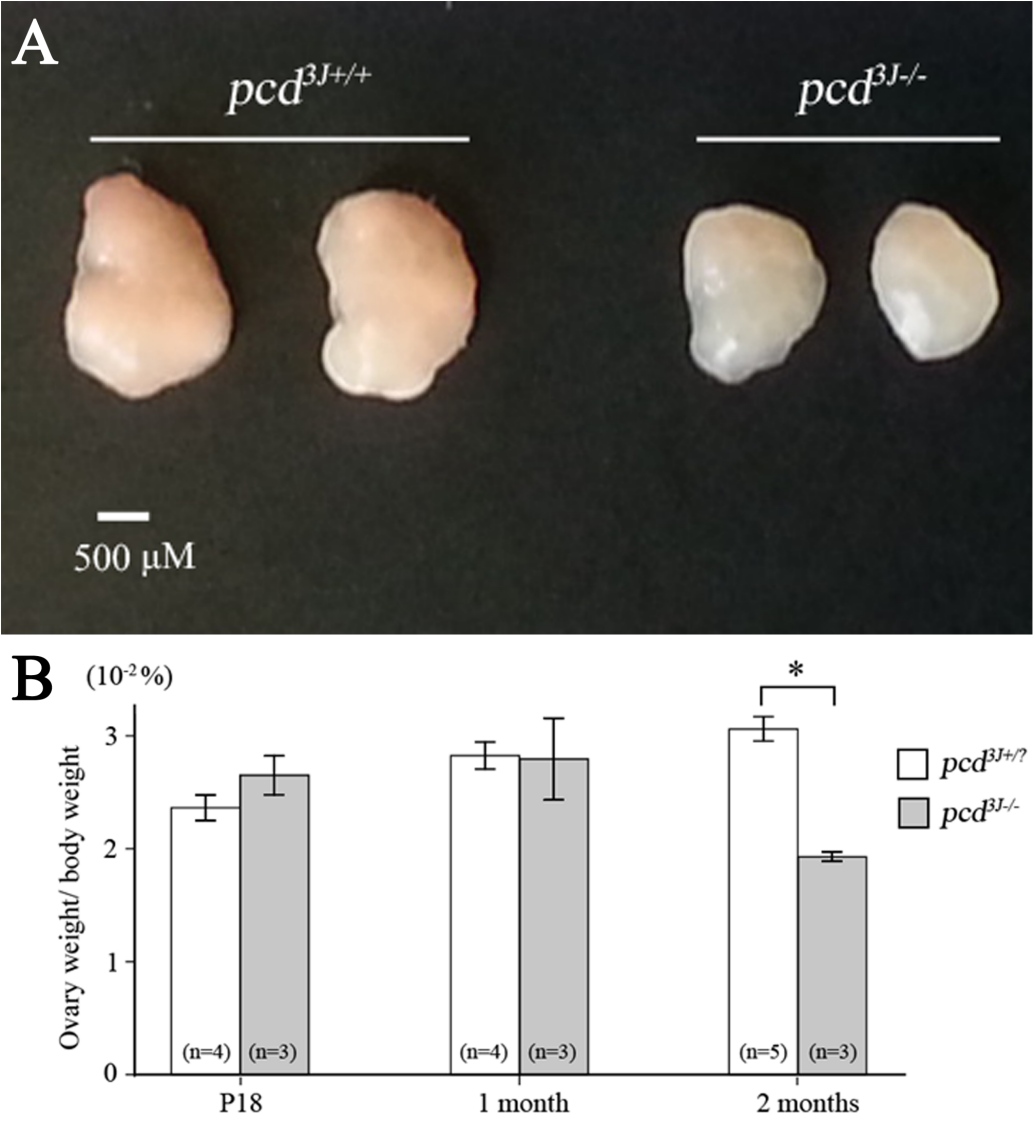
Adult onset ovarian hypoplasia in *pcd* ^*3J-/-*^ mice. (A) Comparison of *pcd* ^*3J+/+*^ and *pcd* ^*3J-/-*^ females revealed a decrease in ovary size at 8 weeks of age. (B) Comparison of relative ovary to body weight differences of *pcd* ^*3J-/-*^ females at P18, 4 weeks old, and 8 weeks old, compared to *pcd* ^*3J+/?*^ mice. The ratio of ovary weight to body weight is presented as the mean ± S.E. Numbers in parentheses indicate the number of mice examined. The difference was statistically significant at 8 weeks of age (*P* < 0.001).

To study the etiology of subfertility in *pcd* ^*3J-/-*^ females, we first analyzed temporal changes in the size of ovaries between *pcd* ^*3J+/+*^ and pcd^*3J-/-*^ females. The size did not show a difference at postpartum 18, which is near the starting point of neuronal degeneration, including Purkinje cell degeneration, in the *pcd* ^*3J-/-*^ mutant cerebellum, but showed gradual hypoplasia afterwards (Fig 1A and 1B). A considerable size difference was observed between wild-type and mutant mice at 8 weeks of age. At 8 weeks of age, the *pcd* ^*3J-/-*^ ovary showed a 37% reduction in the ovary-to-body-weight ratio compared to normal littermates (Fig 1B).

### Decreased number of oocytes with normal appearance after superovulation in *pcd* ^*3J-/-*^ females

**Fig 2:**
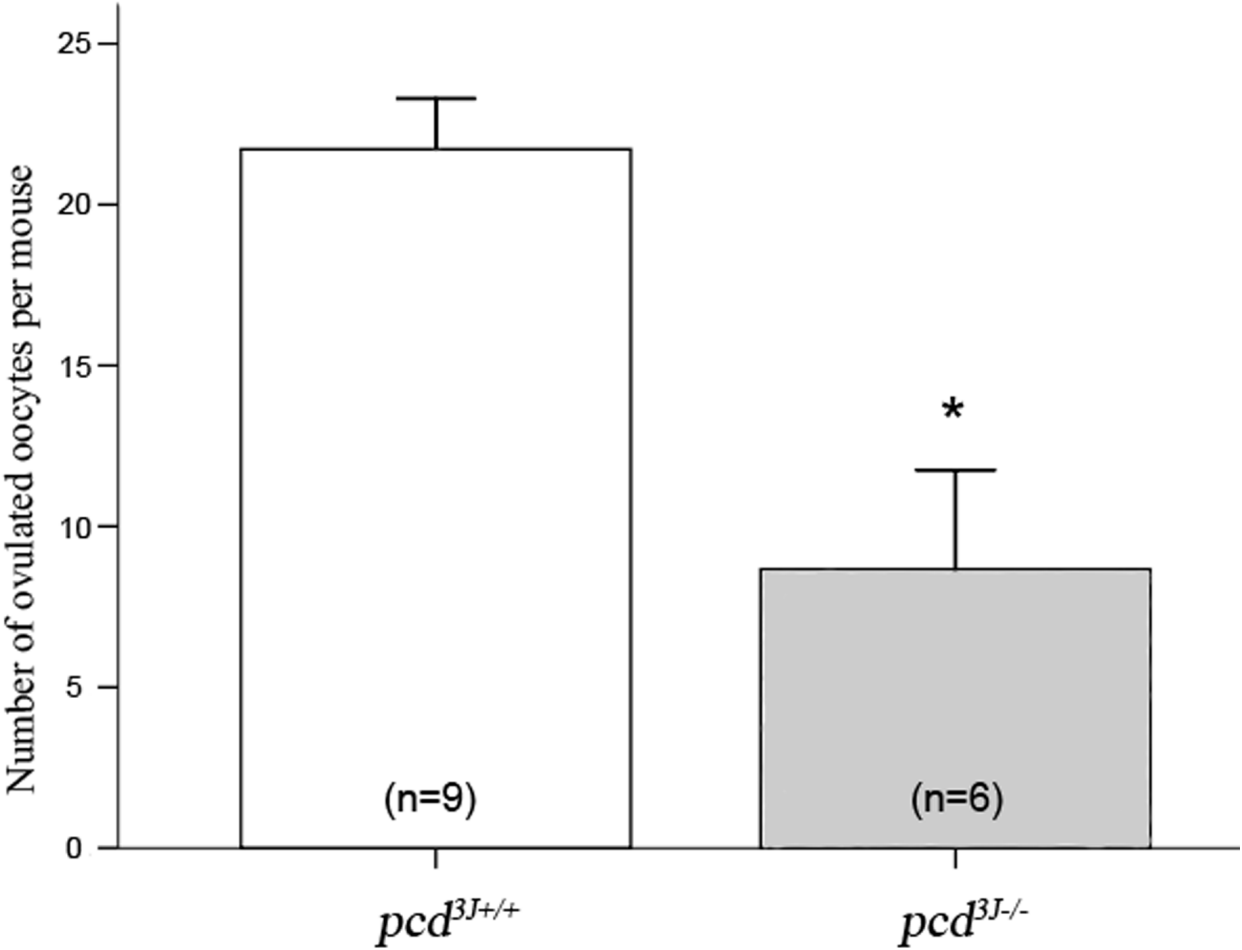
Comparison of the number of collected oocytes after superovulation. The number of oocytes is presented as the mean ± S.E. Numbers in parentheses indicate the number of mice examined. The difference was statistically significant (*P* < 0.001).

The subfertility of *pcd* ^*3J-/-*^ females could either be due to decreased quantity or inferior quality of oocyte production. To test this issue, exogenous gonadotropins were administered to 9 *pcd* ^*3J+/+*^ and 6 *pcd* ^*3J-/-*^ female mice at the age of 8 weeks old to induce superovulation. Ovulated oocytes were recovered from oviducts. The number of oocytes recovered was compared between the two groups. As shown in Fig 2, the number of oocytes in *pcd* ^*3J-/-*^ females was significantly lower than in *pcd* ^*3J+/+*^ females (21.7 ± 3.4 versus 8.0 ± 3.0), indicating that the superficial cause of subfertility in *pcd* ^*3J-/-*^ females is associated with the production of a reduced number of oocytes.

### Abnormal estrus cyclicity in *pcd* ^*3J-/-*^ mice

**Fig 3:**
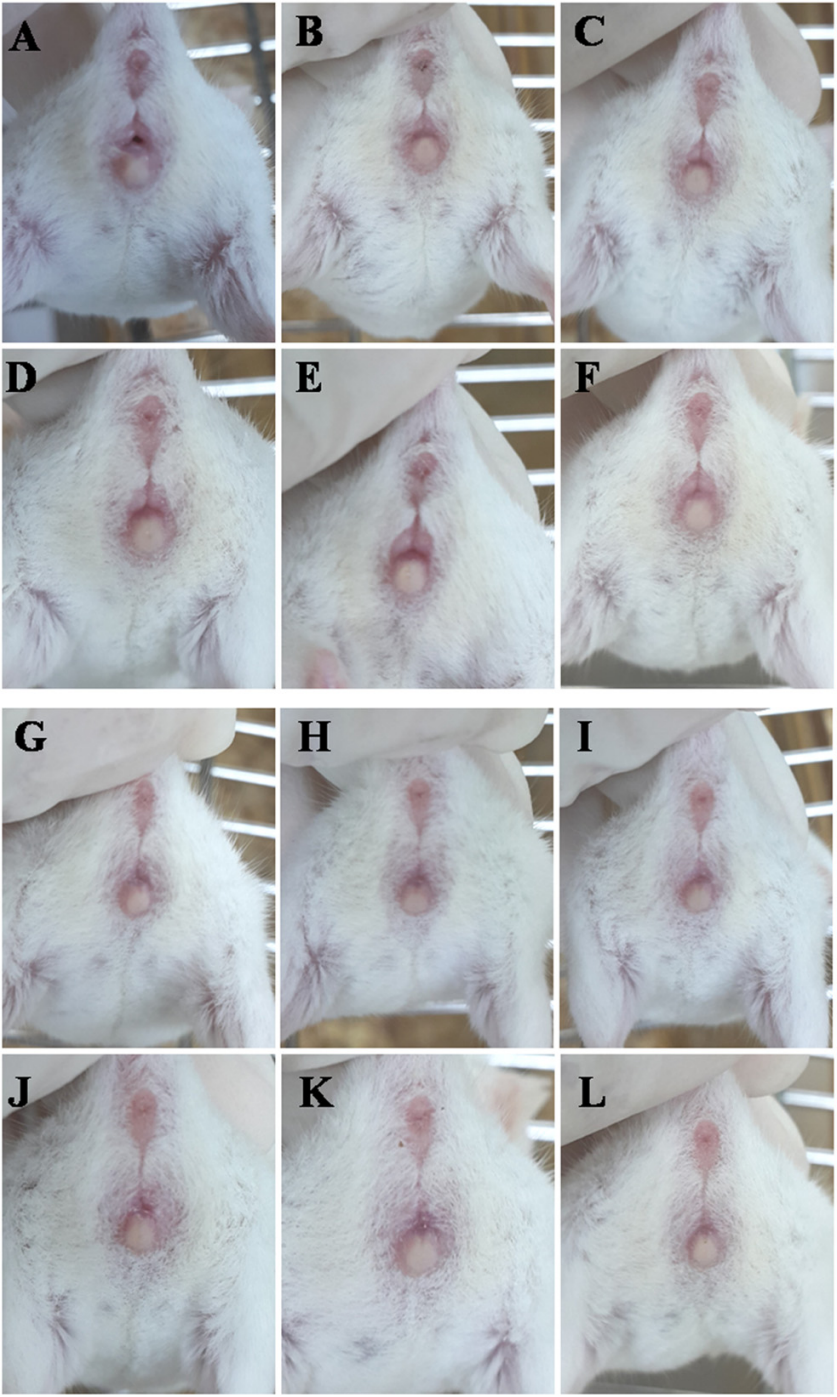
Comparisons of estrous cycles through visual observation of the appearance of the vagina for six consecutive days between *pcd* ^*3J+/+*^ and *pcd* ^*3J-/-*^ female mice. Representative pictures of the appearance of the vagina from *pcd* ^*3J+/+*^ (A, estrus; B, metestrus; C, diestrus; D, proestrus; E, estrous; F, metestrus) and *pcd* ^*3J-/-*^ females (G-L) for six consecutive days. The determination of the specific stage of the estrus cycle was not possible for *pcd* ^*3J-/-*^ and not indicated. Each picture was taken in every 24 hours. No clear signs of the progress in the estrus cycle were observed from *pcd* ^*3J-/-*^ females.

To determine whether *pcd* ^*3J-/-*^ mouse has the normal estrus cycle, we evaluate the appearance of the vagina through visual inspection for *pcd* ^*3J+/+*^ and *pcd* ^*3J-/-*^ mice (n = 6 each) during the weaning to 8 weeks old period. The results showed that *pcd* ^*3J+/+*^ mice showed normal estrus cyclicity with an average length of about 4 days. In contrast, only 2 out of 9 *pcd* ^*3J-/-*^ mice showed active estrus cyclicity. Even for those two, we were able to observe the signs of estrus cyclicity only once and no further vaginal opening or swelling was observed during the period of our observation. Representative pictures from the visual observation of the *pcd* ^*3J+/+*^ and *pcd* ^*3J-/-*^ ovaries during six consecutive days were shown in Fig 3, showing that the signs of the estrus cycle in *pcd* ^*3J-/-*^mice were much weaker or almost undetectable comparing to that of the wild type.

### Reduced number of antral follicles in *pcd* ^*3J-/-*^ ovaries

**Fig 4:**
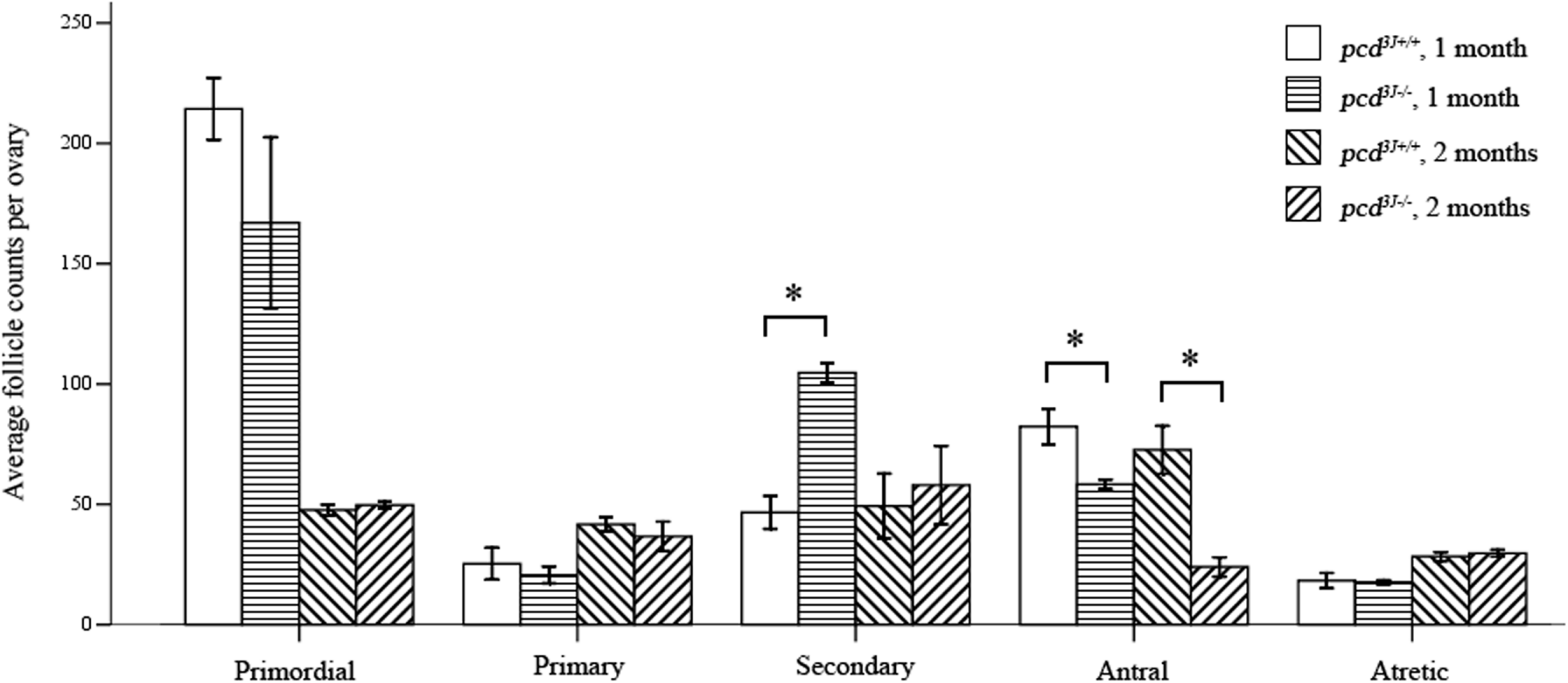
Comparison of the population size of different follicular types between wild *pcd* ^*3J+/+*^ and *pcd* ^*3J-/-*^ females. The average numbers of primordial, primary, secondary, antral, and atretic follicles from a single ovary were determined using three each of 4- and 8-week-old ovaries. Ovaries were serially sectioned, every 10^th^ section was counted, and the total follicle numbers were determined. An increase in secondary follicles and a decrease in antral follicles were observed in *pcd* ^*3J-/-*^ mice compared to the wild type, regardless of age. An asterisk indicates a significant difference (*P* < 0.05).

The decreased number of oocytes following superovulation could be associated with either a decrease in the total number of oocytes in the ovary or to reduced sensitivity of the oocytes to hormonal stimuli. To determine the nature of the abnormalities occurring during oogenesis, sections of *pcd* ^*3J-/-*^ ovaries from 4- and 8-week-old females were examined by H&E staining. Numbers of oocytes in the serial sections of the ovaries were counted. We classified follicles into primordial, primary, secondary, antral, and atretic follicle types. All five types of follicles were observed in both *pcd* ^*3J+/+*^ and *pcd* ^*3J-/-*^ ovaries (Fig 4). Wild-type and *pcd* mutant mice at both 4 and 8 weeks old showed similar numbers of primordial, primary, and atretic follicles. However, the numbers of secondary and antral follicles were consistently different between the two groups. The numbers of secondary and antral follicles in 4-week-old mice, from an analysis of three *pcd* ^*3J+/+*^ and *pcd* ^*3J-/-*^ ovaries, were 47 ± 9 (12.1%) vs. 104 ± 5 (28.8%) and 82 ± 10 (21.2%) vs. 58 ± 2 (15.8%), respectively. The numbers of secondary and antral follicles in 8-week-old mice, from the analysis of three *pcd* ^*3J+/+*^ and *pcd* ^*3J-/-*^ ovaries, were 49 ± 18 (23.3%) vs. 58 ± 20 (33.9%) and 72 ± 12 (34.4%) vs. 24 ± 5 (14.0%), respectively. Although the increase in secondary follicles in 8-week-old mice was not statistically significant, the trend was consistent in both the 4- and 8-week-old groups. The increase in secondary and the decrease in antral follicles in *pcd* ^*3J-/-*^ ovaries over *pcd* ^*3J-/-*^ mice may have resulted from the prevention of secondary follicles from developing into antral follicles. The similar number of primordial follicles observed in *pcd* ^*3J+/+*^ and *pcd* ^*3J-/-*^ mice suggests that the initial production of female germ cells was similar in wild-type and *pcd* mutant mice. The number of primordial follicles (about 2300) observed in our study was consistent to that of a previous report [32].

We also examined the number of oocytes of each follicular type by histological analysis of H&E-stained tissue sections from ovaries of 8-week-old *pcd* ^*3J+/+*^ and *pcd* ^*3J-/-*^ mice after induction of superovulation and immediately before ovulation. This allows the observation of follicular development as a response to gonadotropins. Examination of one *pcd* ^*3J+/+*^ and *pcd* ^*3J-/-*^ ovary showed 14 (14.9%) and 34 (37.3%) secondary oocytes and 44 (46.8%) and 27 (29.6%) antral follicles, respectively. This result was similar to the values observed in ovaries without superovulation, indicating that the lower number of oocytes obtained following superovulation was not due to a deficiency in the ovulatory mechanism itself, but to a decrease in the number of follicles capable of responding to ovulatory signals.

We also examined the association of cell death with the decreased number of ovulated oocytes in *pcd* ^*3J-/-*^ ovaries using a terminal deoxynucleotidyl transferase-mediated deoxyuridinetriphosphate nick end-labeling (TUNEL) assay. We analyzed sections of ovaries from *pcd* ^*3J+/+*^ and *pcd* ^*3J-/-*^ mice at 1 and 2 months of age. However, we were unable to observe a discernable difference in the level of TUNEL-positive signals between *pcd* ^*3J+/+*^ and *pcd* ^*3J-/-*^ ovaries, in addition to the basal level of TUNEL-positive cells which is normally observed in the ovary (S1 Fig), suggesting that subfertility in *pcd* ^*3J-/-*^ mice is not due to the observable level of rapid cell death, but is probably a slowly occurring process.

### The first meiotic division is normal in *pcd* ^*3J-/-*^ females

**Fig 5:**
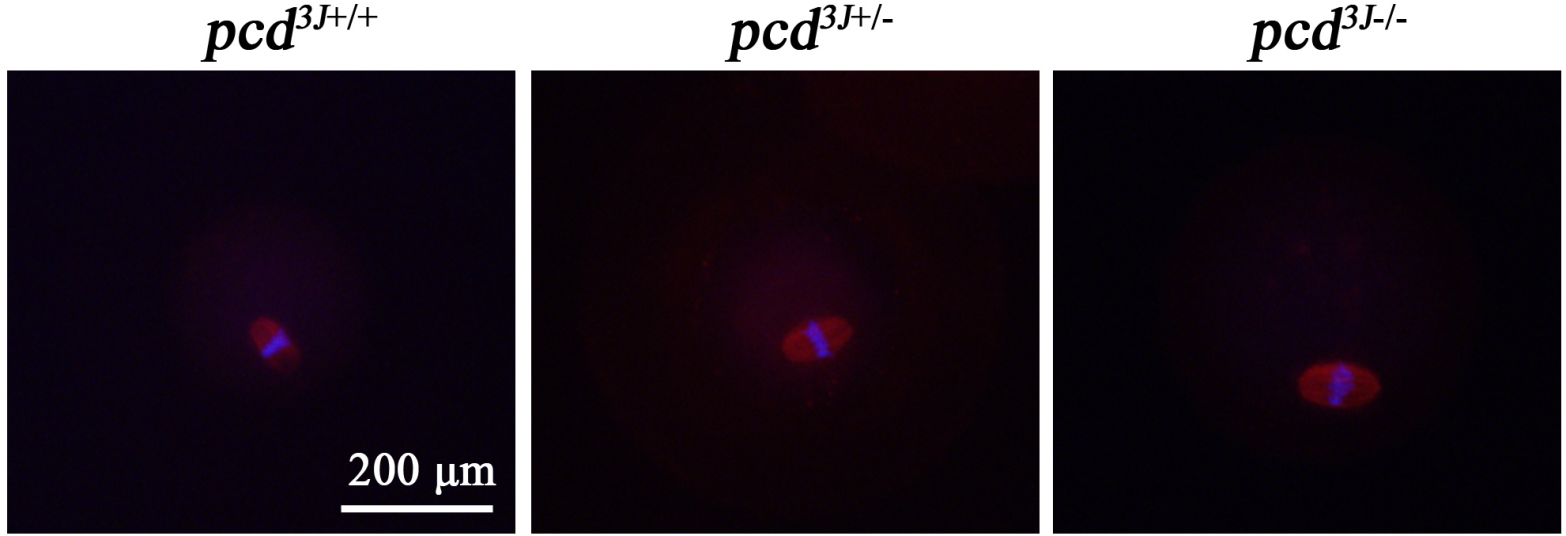
Representative images of ovulated oocytes at the MII stage from *pcd* ^*3J+/+*^, *pcd* ^*3J+/-*^, and *pcd* ^*3J-/-*^females, showing normal chromosomal distribution and spindle formation. Oocytes were stained with anti-α-tubulin primary and Alexa Fluor^®^ 568 anti-mouse IgG secondary antibodies. The nucleus was stained with DAPI. The results from 33, 35, and 30 oocytes from 4 *pcd* ^*3J+/+*^, 3 *pcd* ^*3J+/-*^, and 3 *pcd* ^*3J-/-*^ females, respectively, were analyzed. For *pcd* ^*3J+/+*^ *and pcd^3J+/-^*, only a portion of the oocytes were used for immunocytochemical analysis.

To investigate the mechanism underlying the decreased oocyte release following hormonal stimulation in *pcd* ^*3J-/-*^ females, we examined whether the results of meiosis in *pcd* mutant females are normal. We performed immunohistochemical analysis using an anti-α-tubulin antibody combined with DAPI staining to evaluate the patterns of chromosomal distribution in collected oocytes. Upon ovulation, the majority of oocytes from all three mouse genotypes, *pcd* ^*3J+/+*^, *pcd* ^*3J+/-*^ and *pcd* ^*3J-/-*^, were at the stage of metaphase II arrest when all chromosomes are condensed, connected by spindle fibers, and evenly distributed along the center of the nucleus for all three genotypes (Fig 5), although a few oocytes were still at the GV or GV breakdown stages in all three genotypes. These results indicate that in *pcd* ^3J-/-^ females the meiotic process, up to the metaphase II stage before ovulation, occurs normally.

### Tubulin deglutamylation in *pcd* mice

**Fig 6:**
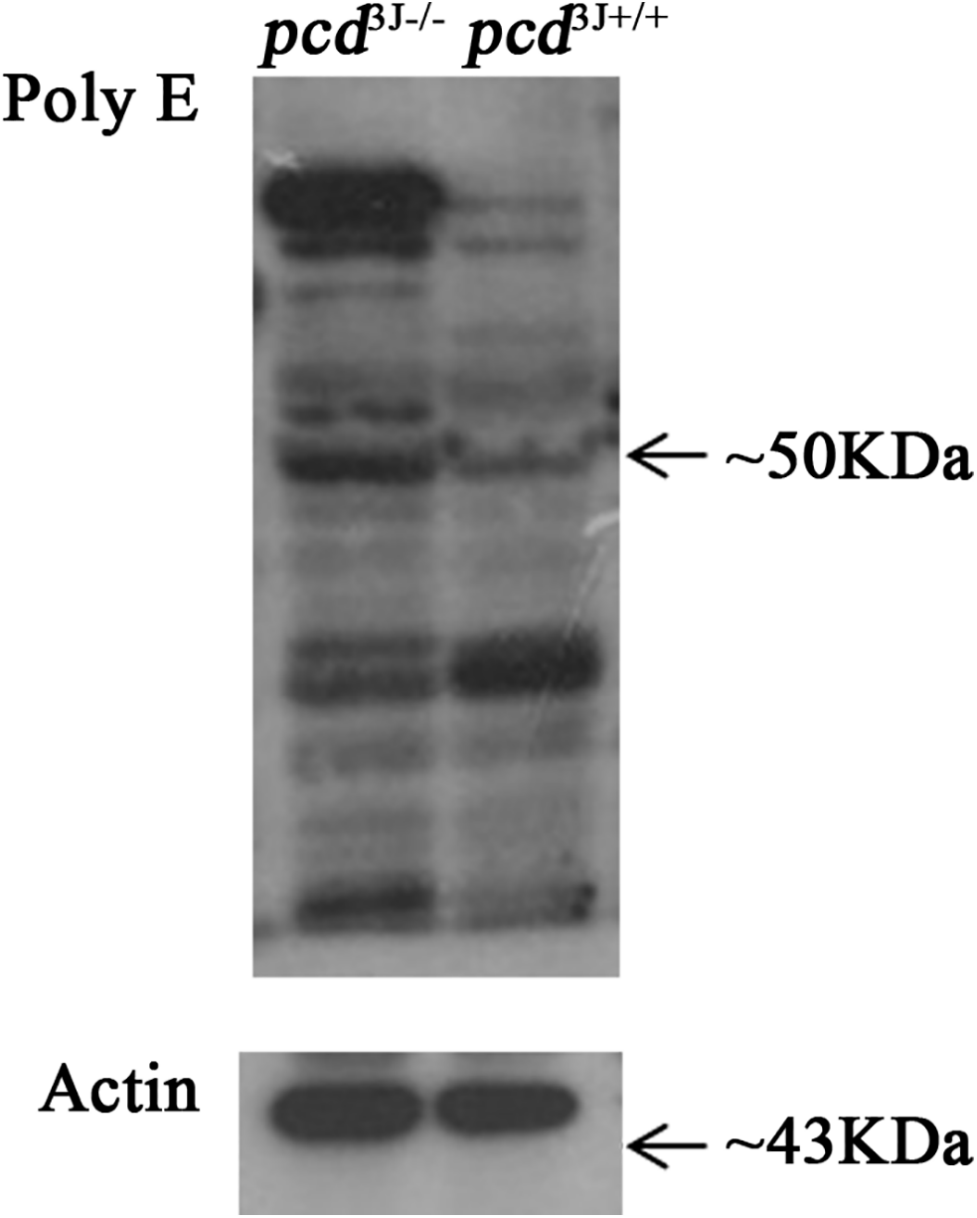
The results of western blot analysis using a polyglutamylation-specific antibody comparing *pcd* ^*3J+/+*^ and pcd^*3J-/-*^ mice. An elevated level of polyglutamylation (polyE) signal was detected from *pcd* ^*3J-/-*^ at ~50 kDa region, which corresponds to tubulin. Actin was used as a quantitative control.

**Fig 7:**
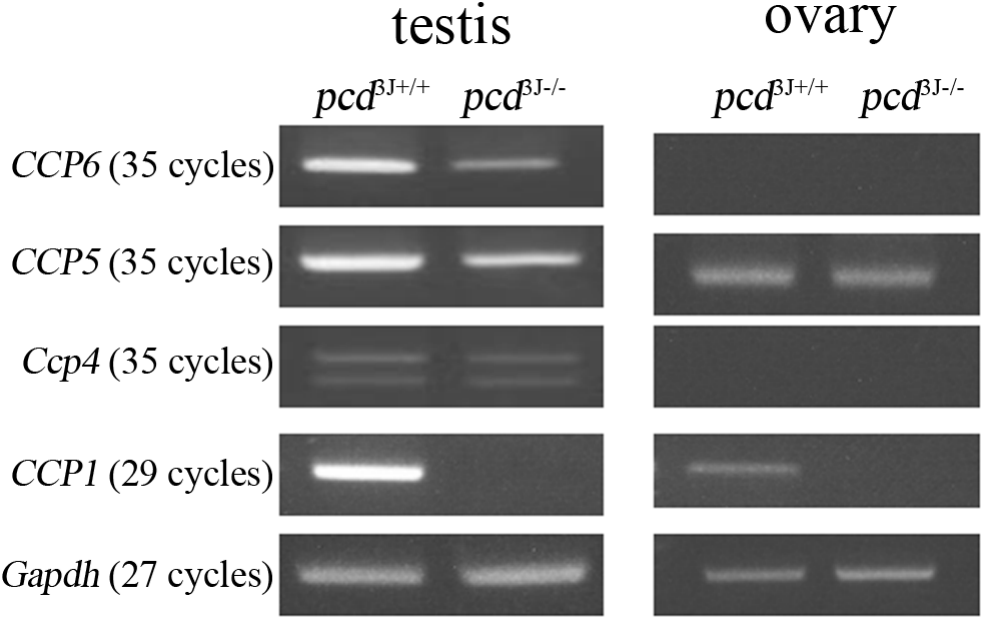
Analysis of expressions of deglutamylases (*CCP1*, *Ccp4*, *CCP5*, *and CCP6*) in testes and ovaries of *pcd* ^*3J+/+*^ and *pcd^3J-/-^* mice using semi-quantitative RT-PCR. The double bands for *Ccp4* indicate two alternatively spliced forms of *Ccp4*. *Gapdh* was used as a quantitative control.

CCP1 is involved in the removal of posttranslational tubulin polyglutamylation [14]. In order to identify the underlying cause of the decreased number of superovulated oocytes, we compared the level of tubulin depolyglutamylation using western blots with polyE antibody between 8-week-old *pcd* ^*3J+/+*^ and *pcd* ^*3J-/-*^ ovaries. Tubulin polyglutamylation was increased in *pcd* ^*3J-/-*^ mice, as expected (Fig 6). *Ccp4* and *CCP6* are functional homologs of *CCP1*, which catalyzes the shortening of glutamate side chains [14]. To explain the decreased levels of glutamylation of the tubulin side chains, we compared the expression levels of *CCP1*, *Ccp4*, *and CCP6* in the ovaries of 8-week-old adult females using semi-quantitative RT-PCR. The results showed that *Ccp4* and *CCP6* were not expressed in ovaries of both *pcd* ^*3J+/+*^ and *pcd* ^*3J-/-*^ mice (Fig 7), which is consistent with the previous finding that the cerebellum of *pcd* ^*3J-/-*^ mice expressing very low levels of *Ccp4* and *CCP6* was unable to compensate the lack of *CCP1*, leading to the death of Purkinje cells in the cerebellum [14]. Therefore, the lack of CCP1 activity in *pcd* ^*3J-/-*^ mice resulted in decreased tubulin deglutamylation in ovaries, which could be either an associated phenotype or the direct cause of the decreased number of antral follicles.

### Expression of *CCP1*, *Ccp4*, *and CCP6* in GV oocytes and granulosa cells

**Fig 8:**
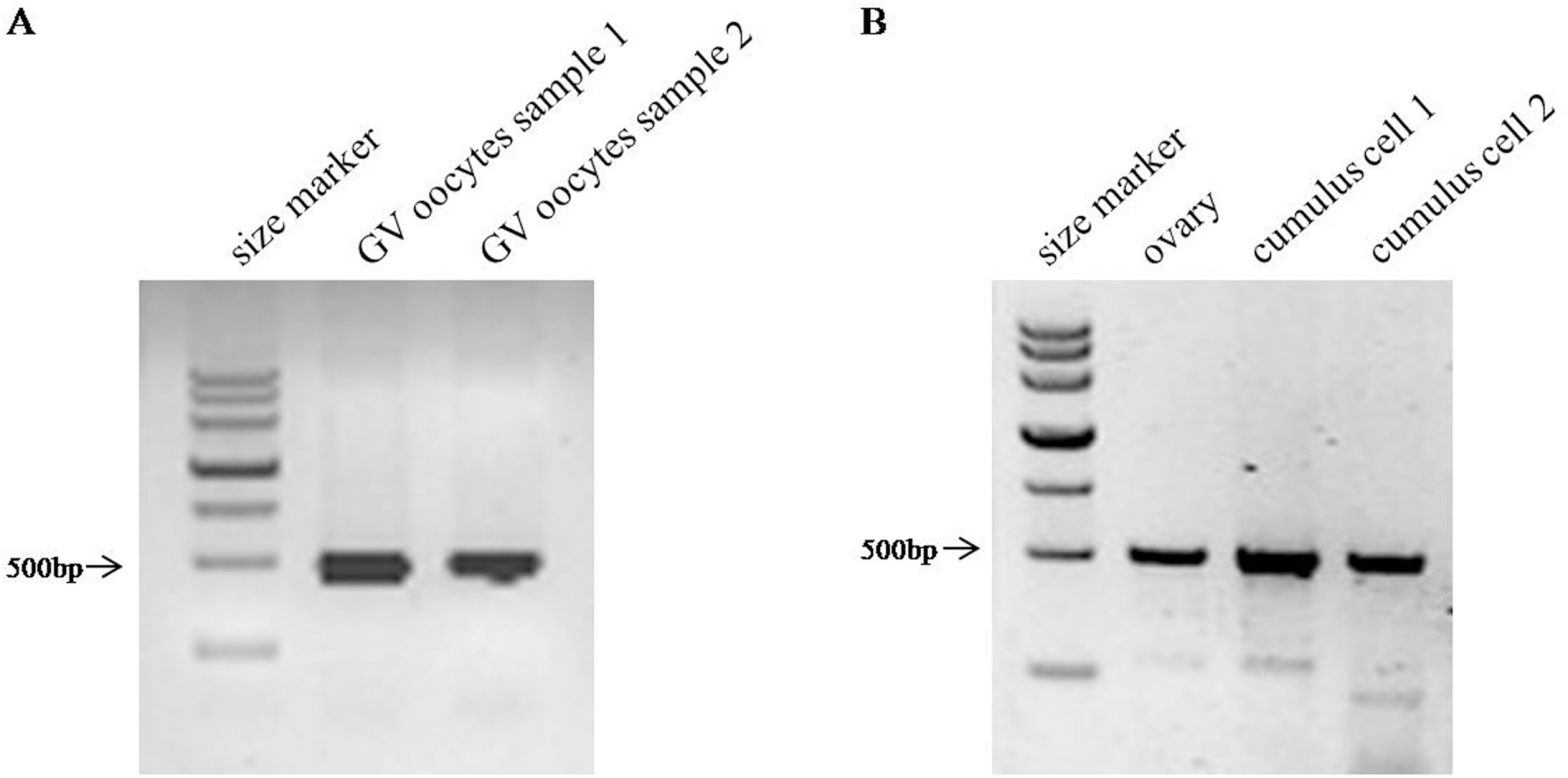
Analysis of *CCP1* expression in GV oocytes and granulosa cells using semi-quantitative RT-PCR. Negative controls did not produce any products and are not shown.

To address the mechanism underlying the decreased number of antral follicles in *pcd* ^*3J-/-*^ ovaries, we compared the mRNA expression of *CCP* ***1*,** *Ccp4*, *and CCP6* against specific cell types in the ovary, GV oocytes, and granulosa cells. We found that both GV oocytes and granulosa cells expressed *CCP* ***1*** (Fig 8). Consistent with the results shown in Fig 7, there was no expression of *Ccp4* or *CCP6* in GV oocytes or granulosa cells (data not shown), indicating that abnormalities in either GV oocytes or granulosa cells themselves could be the cause of the lower number of antral follicles, although the effects of the lack of *CCP1* on the activities of each cell type require further study.

## Discussion

CCP1 is known to act as a functional cytosolic carboxypeptidase that removes Glu residues from the C-terminus of α-tubulin and the side chains of both α- and β-tubulin. However, it is somewhat unclear whether CCP1, in its role as a tubulin-processing enzyme, is directly responsible for the broad cellular changes observed in numerous previous studies on *pcd* mice, especially considering that the abnormalities begin to occur around day 20, when organismal development is largely complete [1, 33]. Other cytosolic proteins that require depolyglutamylation modification may also be involved [14, 34]. Therefore, the complete mechanism underlying *pcd* phenotypes remains to be revealed.

In this study, we reported the results of detailed phenotypic and cellular analyses of the factors underlying the subfertility of *pcd* ^*3J-/-*^ females. We found that the lower fecundity of *pcd* mutant females was caused by a decreased number of antral follicles and showed that CCP1 is essential for follicular development, especially from secondary to antral follicles, providing another phenotype for the *CCP1* mutation, which differs from previously characterized cell death phenotypes in *pcd* ^*3J-/-*^ mice, such as degeneration in regions of the brain, eyes, and testes [8, 33].

The antral follicle count (AFC) is considered to be a reliable and non-invasive method to determine the ovarian reserve and is a good indicator of the size of the remaining primordial pool in women with proven natural fertility [35]. A low AFC has been described in women during the perimenopausal period [36, 37], and also in young infertile and subfertile women affected by premature ovarian failure [38, 39]. Moreover, low AFC has also been noted in the ovaries of mutant mice lacking Akt1 [40], resulting in female subfertility. *Pcd* ^*3J-/-*^ mice also show low AFC, and our findings indicated that CCP1 plays an important role in the regulation of the growth and maturation of ovarian follicles. As a possible reason for the low AFC, we compared the difference in the transcript level of anti-mullerian hormone (AMH) between wild type and *pcd* mutant ovaries. However, the expression level was similar between wild type and normal mice (data not shown), suggesting that not AMH but other reasons caused the defects in the follicular development in *pcd* ^*3J-/-*^ ovaries. In *Akt1* ^*-/-*^, in response to exogenous gonadotropins, the number of secondary follicles was significantly increased without an increase in the number of antral follicles; this phenotype is similar in *pcd* ^*3J-/-*^ ovaries, as described in our results. Furthermore, the results of IVF experiments between *pcd* ^*3J+/+*^ and *pcd* ^*3J-/-*^ oocytes using sperm from *pcd* ^*3J+/+*^ showed no difference in oocyte development to blastocysts after fertilization (data not shown), suggesting that CCP1 plays an important role in follicular development but the developmental capacity of ovulated oocytes from *pcd* ^*3J-/-*^ is not affected by the lack of CCP1. This explains why *pcd* ^*3J-/-*^ females sometimes produce a small number of pups when they were mated to wild type males, which could be described as subfertility.

The consequences of the failure of microtubule deglutamylation caused by a lack of *CCP1* have not been clearly addressed, although recent studies have reported an increase in polyglutamylation in the microtubules of *pcd* mutant mice [13, 14]. The spindle is primarily composed of microtubules, which are polarized filaments consisting of α/β-tubulin heterodimers arranged in a head-to-tail configuration within protofilaments [41]. The mitotic spindle of HeLa cells is polyglutamylated, but both the mitotic and meiotic spindles of germ cells are monoglutamylated [42]. Spindles consisting of polyglutamylated microtubules might show adversely influenced functioning in somatic cells, but further studies are required to address this question.

In our previous study on spermatogenesis in *pcd* ^*3J-/-*^ males [8], we were unable to examine the process of meiotic cell division due to the experimental limitations of analyzing condensed male germ cells. In this study, using oocytes to analyze meiosis allowed us to analyze the integrity of the meiotic spindle and showed that there was no observable abnormality in the function of the meiotic spindle in metaphase II oocytes, as shown in Fig 5. This is consistent with other reports that glutamylation is not essential for the assembly and function of microtubules, although it affects the assembly and function of a subset of microtubule-based organelles [43]. Therefore, polyglutamylation of microtubules may affect cellular events other than spindle formation, and thus may be an indicator for defects in *CCP1*.

It has been shown that *CCP6* compensates for the absence of *CCP1* in the cortex of *pcd* mice, although it is present at levels too low to achieve complete compensation in the cerebellum, resulting in tubulin hyperglutamylation and Purkinje cell death in the cerebellum [14]. If the lack of salvage pathways is the underlying cause of the tissue specificity of the observed defects of *pcd* ^*3J-/-*^ mice, the expression pattern of CCP1-related proteins should not overlap with those of either *Ccp4* or *CCP6* in the affected tissues. Therefore, we examined the expression patterns of *CCP1*, *Ccp4*, and *CCP6* in germ cell-producing organs, i.e. the ovary and testis, of *pcd* ^*3J-/-*^ males and females. The lack of *Ccp4* and *CCP6* expression in ovaries was consistent with the previous explanation for the tissue-specific death of Purkinje cells that does not affect neurons in the neocortex. *CCP5* was also expressed in both ovaries and testes (Fig 7). However, expression of *CCP6* was high in the testis (Fig 7), which does not support the hypothesis, because this is the affected tissue in *pcd* ^*3J-/-*^ mice [1, 8, 44, 45]. This discrepancy may be because *CCP1* and *CCP6* are expressed by different cells in testes, or there may be other factors, which have yet to be identified. For example, there may be other targets of CCP1 that play an essential role in germ cell development in the testis.

It is interesting that *pcd* ^*3J-/-*^ mice show abnormalities in the development of both male and female germ cells. This prompted us to consider whether a defect in a germ cell-specific pathway may be common to both males and females. In *pcd* ^*3J-/-*^ testes, apoptosis begins with pachytene spermatocytes, indicating that the early periods of spermatogenesis are affected; only a few differentiated spermatids with morphological abnormalities were present [8]. In the ovaries, although fewer oocytes were ovulated, they were functionally intact. The number of oocytes after super-ovulation could be varied depending on stages of the estrous cycle because animals are at uncertain stages of the estrous cycle. The sizes of ovaries were similar in one month but become clearly hypoplasic in two month between wild type and mutant mice. Especially, although there showed no clear difference in numbers of primordial and primary follicles both in one month and two month old ovaries, dramatic reduction in the number of antral follicles was observed especially in two month old *pcd* mutant ovaries (Fig 4). Considering that the progression of the estrus cycle is abnormal in *pcd* mutant mice (Fig 3) and that the variation in the number of oocytes among multiple mice (n = 15) after superovulation is relatively limited, we think that the number of produced oocytes after superovulation in *pcd* mutant females in our experiment is close to the true value. More importantly, all results on the analyses of subfertility in *pcd* mutant mice were consistent.

In addition, the early phase of meiosis is normal, in contrast to testes, suggesting that the causes of defects in germ cell development in male and female *pcd* ^*3J-/-*^ mice may differ. Comparing the underlying mechanism for adult onset neuronal death and defects in germ cell development using *pcd* ^*3J-/-*^ mice could provide new insight into the function of CCP1.

## Supporting Information

S1 Fig Comparison of the terminal deoxynucleotidyl transferase-mediated deoxyuridinetriphosphate nick end-labeling (TUNEL) assay between *pcd* ^*3J+/+*^ and *pcd* ^*3J-/-*^ ovaries. Fig 1A and 1C are from *pcd* ^*3J+/+*^ and *pcd* ^*3J-/-*^ mice at one month old. Fig 1B and 1D are from *pcd* ^*3J+/+*^and *pcd* ^*3J-/-*^ mice at two months old. Fig 1G and 1H are positive and negative for the TUNEL assay, respectively.
(TIF)

S1 Table The summary of reported *CCP1* alleles (DOC)

S2 Table Comparison of body weights between *pcd* ^*3J+/+*^ and *pcd* ^*3J-/-*^ female mice at 8 weeks old. (DOC)

## References

[1] Mullen RJ, Eicher EM, Sidman RL. Purkinje cell degeneration, a new neurological mutation in the mouse. Proc Natl Acad Sci U S A. 1976;73(1):208–12. 1061118 10.1073/pnas.73.1.208 PMC335870

[2] Landis SC, Mullen RJ. The development and degeneration of Purkinje cells in *pcd* mutant mice. J Comp Neurol. 1978;177:125–44. 200636 10.1002/cne.901770109

[3] Blanks JC, Mullen RJ, LaVail MM. Retinal degeneration in the *pcd* cerebellar mutant mouse. II. Electron microscopic analysis. J Comp Neurol. 1982;212(3):231–46. 10.1002/cne.902120303 . 7153375

[4] LaVail MM, Blanks JC, Mullen RJ. Retinal Degeneration in the *pcd* Cerebellar Mutant Mouse. I. Light Microscopic and Autoradiographic Analysis. J Comp Neurol. 1982;2l2:217–30. 10.1002/cne.902120302 7153374

[5] Greer CA, Shepherd GM. Mitral cell degeneration and sensory function in the neurological mutant mouse Purkinje cell degeneration (*PCD*). Brain Res. 1982;235(1):156–61. . 7188319 10.1016/0006-8993(82)90206-2

[6] O'Gorman S, Sidman RL. Degeneration of Thalamic Neurons in “Purkinje Cell Degeneration” Mutant Mice. I. Distribution of Neuron Loss. J Comp Neurol. 1985;234:277–97. 3988985 10.1002/cne.902340302

[7] O'Gorman S. Degeneration of Thalamic Neurons in “Purkinje Cell Degeneration” Mutant Mice. 11. Cytology of Neuron Loss. J Comp Neurol. 1985;234:298–316. 3988986 10.1002/cne.902340303

[8] Kim N, Xiao R, Choi H, Jo H, Kim JH, Uhm SJ, et al Abnormal Sperm Development in *pcd* ^*(3J)-/-*^ Mice: the Importance of Agtpbp1 in Spermatogenesis. Mol Cells. 2011;31(1):39–48. 10.1007/s10059-011-0002-1 . 21110128 PMC3906870

[9] Fernandez-Gonzalez A, La Spada AR, Treadaway J, Higdon JC, Harris BS, Sidman RL, et al Purkinje cell degeneration (*pcd*) phenotypes caused by mutations in the axotomy-induced gene, Nna1. Science. 2002;295(5561):1904–6. 10.1126/science.1068912 . 11884758

[10] Kalinina E, Biswas R, Berezniuk I, Hermoso A, Aviles FX, Fricker LD. A novel subfamily of mouse cytosolic carboxypeptidases. Faseb Journal. 2007;21(3):836–50. 10.1096/fj.06-7329com . 17244818

[11] Rodriguez de la Vega Otazo M, Lorenzo J, Tort O, Aviles FX, Bautista JM. Functional segregation and emerging role of cilia-related cytosolic carboxypeptidases (CCPs). Faseb J 2013;27(2):424–31. 10.1096/fj.12-209080 . 23085998

[12] Wu HY, Wang TY, Li LY, Correia K, Morgan JI. A structural and functional analysis of Nna1 in Purkinje cell degeneration (*pcd*) mice. Faseb J 2012;26(11):4468–80. 10.1096/Fj.12-205047 . 22835831 PMC3475255

[13] Berezniuk I, Vu HT, Lyons PJ, Sironi JJ, Xiao H, Burd B, et al Cytosolic Carboxypeptidase 1 Is Involved in Processing alpha- and beta-Tubulin. J Biol Chem. 2012;287(9):6503–17. 10.1074/jbc.M111.309138 . 22170066 PMC3307270

[14] Rogowski K, van Dijk J, Magiera MM, Bosc C, Deloulme JC, Bosson A, et al A family of protein-deglutamylating enzymes associated with neurodegeneration. Cell. 2010;143(4):564–78. 10.1016/j.cell.2010.10.014 . 21074048

[15] Berezniuk I, Sironi JJ, Wardman J, Pasek RC, Berbari NF, Yoder BK, et al Quantitative Peptidomics of Purkinje Cell Degeneration Mice. Plos One. 2013;8(4). doi: ARTN e60981 10.1371/journal.pone.0060981 . PMC3620535 23593366

[16] Tanco S, Tort O, Demol H, Aviles FX, Gevaert K, Van Damme P, et al C-terminomics Screen for Natural Substrates of Cytosolic Carboxypeptidase 1 Reveals Processing of Acidic Protein C termini. Mol Cell Proteomics. 2015;14(1):177–90. 10.1074/mcp.M114.040360 . 25381060 PMC4288253

[17] Valero J, Berciano MT, Weruaga E, Lafarga M, Alonso JR. Pre-neurodegeneration of mitral cells in the *pcd* mutant mouse is associated with DNA damage, transcriptional repression, and reorganization of nuclear speckles and Cajal bodies. Mol Cell Neurosci. 2006;33(3):283–95. 10.1016/j.mcn.2006.08.002 . 16978877

[18] Baltanas FC, Casafont I, Lafarga V, Weruaga E, Alonso JR, Berciano MT, et al Purkinje Cell Degeneration in *pcd* Mice Reveals Large Scale Chromatin Reorganization and Gene Silencing Linked to Defective DNA Repair. J Biol Chem. 2011;286(32):28287–302. 10.1074/jbc.M111.246041 . 21700704 PMC3151073

[19] Kyuhou S, Kato N, Gemba H. Emergence of endoplasmic reticulum stress and activated microglia in Purkinje cell degeneration mice. Neurosci Lett. 2006;396(2):91–6. 10.1016/j.neulet.2005.11.023 . 16356646

[20] Bäurle J, Grüsser-Cornehls U. Axonal torpedoes in cerebellar Purkinje cells of two normal mouse strains during aging. Acta Neuropathol. 1994;88:237–45. 7810294 10.1007/BF00293399

[21] Chakrabarti L, Zahra R, Jackson SM, Kazemi-Esfarjani P, Sopher BL, Mason AG, et al Mitochondrial Dysfunction in NnaD Mutant Flies and Purkinje Cell Degeneration Mice Reveals a Role for Nna Proteins in Neuronal Bioenergetics. Neuron. 2010;66(6):835–47. 10.1016/j.neuron.2010.05.024 . 20620870 PMC3101252

[22] Berezniuk I, Sironi J, Callaway MB, Castro LM, Hirata IY, Ferro ES, et al CCP1/Nna1 functions in protein turnover in mouse brain: Implications for cell death in Purkinje cell degeneration mice. Faseb Journal. 2010;24(6):1813–23. 10.1096/Fj.09-147942 . 20061535 PMC2874472

[23] Li J, Gu X, Ma Y, Calicchio ML, Kong D, Teng YD, et al Nna1 mediates Purkinje cell dendritic development via lysyl oxidase propeptide and NF-kappaB signaling. Neuron. 2010;68(1):45–60. 10.1016/j.neuron.2010.08.013 . 20920790 PMC4457472

[24] Campbell PK, Waymire KG, Heier RL, Sharer C, Day DE, Reimann H, et al Mutation of a novel gene results in abnormal development of spermatid flagella, loss of intermale aggression and reduced body fat in mice. Genetics. 2002;162(1):307–20. 12242242 10.1093/genetics/162.1.307 PMC1462267

[25] Ikegami K, Heier RL, Taruishi M, Takagi H, Mukai M, Shimma S, et al Loss of alpha-tubulin polyglutamylation in ROSA22 mice is associated with abnormal targeting of KIF1A and modulated synaptic function. P Nati Acad Sci USA. 2007;104(9):3213–8. 10.1073/pnas.0611547104 . PMC1802010 17360631

[26] Janke C, Rogowski K, van Dijk J. Polyglutamylation: a fine-regulator of protein function? 'Protein Modifications: beyond the usual suspects' review series. EMBO Rep. 2008;9(7):636–41. 10.1038/embor.2008.114 18566597 PMC2475320

[27] Xiao R, Park Y, Dirisala VR, Zhang YP, Um SJ, Lee HT, et al Identification of genes differentially expressed in wild type and Purkinje cell degeneration mice. Mol Cells. 2005;20(2):219–27. . 16267396

[28] Champlin AK, Dorr DL, Gates AH. Determining the stage of the estrous cycle in the mouse by the appearance of the vagina. Biol Reprod. 1973;8(4):491–4. . 4736343 10.1093/biolreprod/8.4.491

[29] Bradford MM. A rapid and sentitive method for the quantitation of microgram quantities of protein utilizing the principle of protein-dte binding Anal Biochem. 1976;72:248–54. 942051 10.1016/0003-2697(76)90527-3

[30] Shang Y, Li B, Gorovsky MA. Tetrahymena thermophila contains a conventional gamma-tubulin that is differentially required for the maintenance of different microtubule-organizing centers. J Cell Biol. 2002;158(7):1195–206. 10.1083/jcb.200205101 12356864 PMC2173235

[31] Britt KL, Drummond AE, Cox VA, Dyson M, Wreford NG, Jones MEE, et al An age-related ovarian phenotype in mice with targeted disruption of the Cyp 19 (aromatase) gene. Endocrinology. 2000;141(7):2614–23. 10.1210/en.141.7.2614 . 10875266

[32] Johnson J, Canning J, Kaneko T, Pru JK, Tilly JL. Germline stem cells and follicular renewal in the postnatal mammalian ovary. Nature. 2004;428(6979):145–50. 10.1038/nature02316 . 15014492

[33] Wang TY, Morgan JI. The Purkinje cell degeneration (*pcd*) mouse: An unexpected molecular link between neuronal degeneration and regeneration. Brain Res. 2007;1140:26–40. 10.1016/j.brainres.2006.07.065 . 16942761

[34] Van Dijk J, Miro J, Strub JM, Lacroix B, Van Dorsselaer A, Edde B, et al Polyglutamylation is a post-translational modification with a broad range of substrates. J Biol Chem. 2008;283(7):3915–22. 10.1074/jbc.M705813200 . 18045879

[35] Hansen KR, Hodnett GM, Knowlton N, Craig LB. Correlation of ovarian reserve tests with histologically determined primordial follicle number. Fertility and sterility. 2011;95(1):170–5. 10.1016/j.fertnstert.2010.04.006 . 20522327

[36] Hansen KR, Knowlton NS, Thyer AC, Charleston JS, Soules MR, Klein NA. A new model of reproductive aging: the decline in ovarian non-growing follicle number from birth to menopause. Hum Reprod. 2008;23(3):699–708. 10.1093/humrep/dem408 . 18192670

[37] Broekmans FJ, Soules MR, Fauser BC. Ovarian Aging: Mechanisms and Clinical Consequences. Endocr Rev. 2009;30(5):465–93. 10.1210/Er.2009-0006 . 19589949

[38] De Vos M, Devroey P, Fauser BCJM. Primary ovarian insufficiency. Lancet. 2010;376(9744):911–21. 10.1016/S0140-6736(10)60355-8 . 20708256

[39] Monget P, Bobe J, Gougeon A, Fabre S, Monniaux D, Dalbies-Tran R. The ovarian reserve in mammals: A functional and evolutionary perspective. Mol Cell Endocrinol. 2012;356(1–2):2–12. 10.1016/j.mce.2011.07.046 . 21840373

[40] Brown C, LaRocca J, Pietruska J, Ota M, Anderson L, Smith SD, et al Subfertility caused by altered follicular development and oocyte growth in female mice lacking PKB alpha/Akt1. Biol Reprod. 2010;82(2):246–56. 10.1095/biolreprod.109.077925 . 19794155 PMC6058744

[41] Walczak CE, Heald R. Mechanisms of mitotic spindle assembly and function. Int Rev Cytol. 2008;265:111–58. 10.1016/S0074-7696(07)65003-7 . 18275887

[42] Kann ML, Soues S, Levilliers N, Fouquet JP. Glutamylated tubulin: diversity of expression and distribution of isoforms. Cell motility and the cytoskeleton. 2003;55(1):14–25. 10.1002/cm.10107 . 12673595

[43] Wloga D, Rogowski K, Sharma N, Van Dijk J, Janke C, Edde B, et al Glutamylation on alpha-tubulin is not essential but affects the assembly and functions of a subset of microtubules in Tetrahymena thermophila. Eukaryot Cell. 2008;7(8):1362–72. 10.1128/EC.00084-08 18586949 PMC2519764

[44] Krulewski TF, Neumann PE, Gordon JW. Insertional mutation in a transfenic mouse allelic with purkinje cell degeneration. P Nati Acad Sci USA. 1989;86:3709–12. 10.1073/pnas.86.10.3709 PMC287209 2726749

[45] Handel M A., Dawson M. Effects on spermiogenesis in the mouse of a male sterile neurological mutation, purkinje cell degeneration. Gamete Res. 1981;4:185–92.

