# Supplementary figures and images for: Lack of Cytosolic Carboxypeptidase 1 Leads to Subfertility due to the Reduced Number of Antral Follicles in pcd3J-/- Females
Source: PLoS One. 2015 Oct 9;10(10):e0139557. doi: 10.1371/journal.pone.0139557 (PMC4599934; doi:10.1371/journal.pone.0139557)

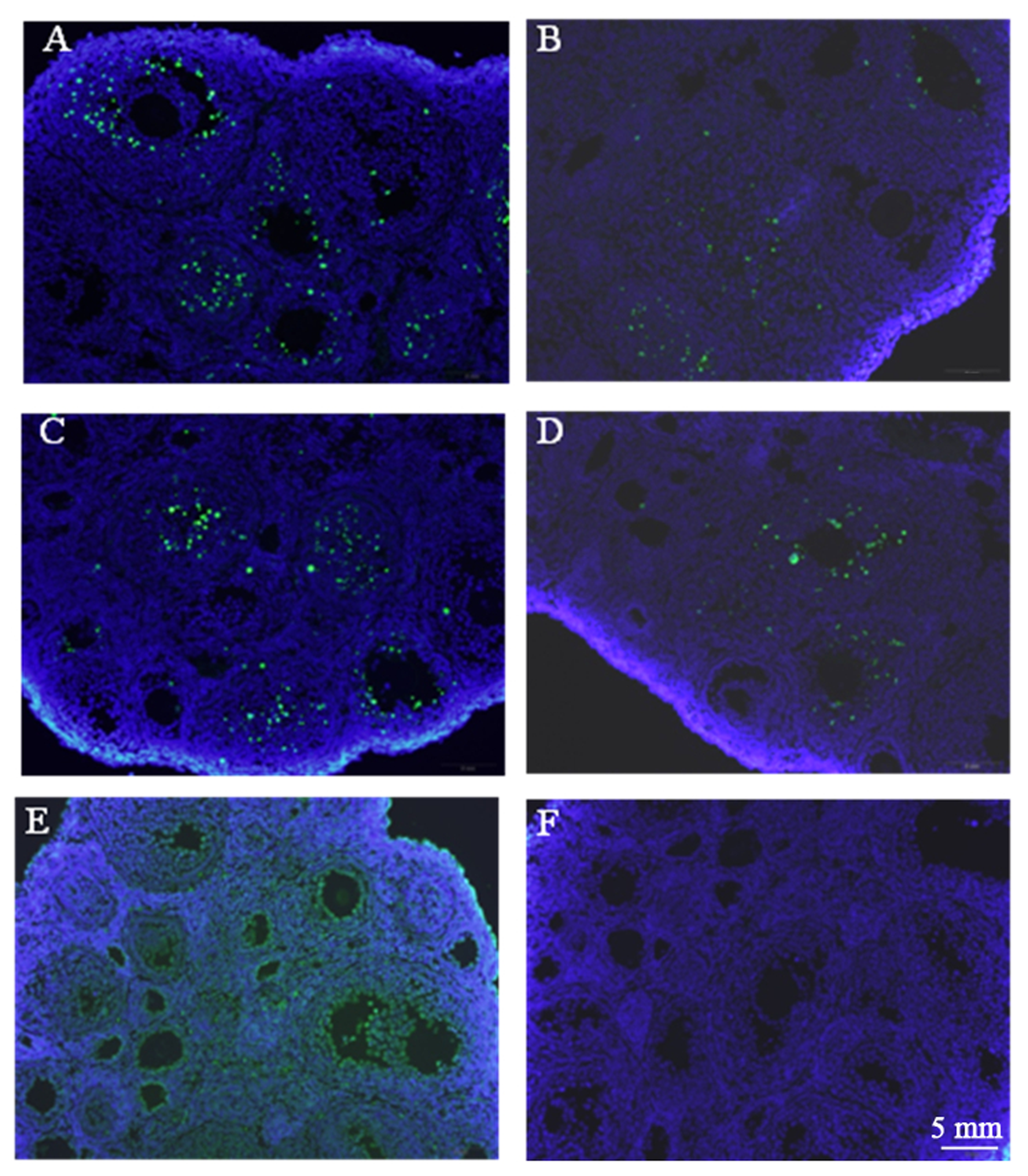

Supplement: S1 Fig — Fig 1A and 1C are from pcd 3J+/+ and pcd 3J-/- mice at one month old. Fig 1B and 1D are from pcd 3J+/+and pcd 3J-/- mice at two months old. Fig 1G and 1H are positive and negative for the TUNEL assay, respectively. (TIF) [file pone.0139557.s001.tif]
