# Supplementary material for: Lack of Cytosolic Carboxypeptidase 1 Leads to Subfertility due to the Reduced Number of Antral Follicles in pcd3J-/- Females
Source: PLoS One. 2015 Oct 9;10(10):e0139557. doi: 10.1371/journal.pone.0139557 (PMC4599934; doi:10.1371/journal.pone.0139557)
Supplement: S1 Table — (DOC) [file pone.0139557.s002.doc]

| Supplementary Table 1. The summary of reported *CCP1* alleles | | | |
| --- | --- | --- | --- |
| Allele name | Origin | Phenotypes and morphological changes | Genetic mutations in Nna1 |
| *CCP1*^pcd^ | Spontaneous | Ataxia; degeneration of cerebellar Purkinje cells and granule cells; degeneration of thalamus neurons, olfactory bulb mitral cells; degeneration of photoreceptor cells; male infertility, female partial fertility; degeneration of sperm. Very low mRNA and protein levels. | Unknown (possibly in regulatory region) |
| *CCP1*^pcd-2J^ | Spontaneous | Presumptive hypomorphic allele with slower onset ataxia and some male infertility. Low mRNA levels. | Insertion between exons 14 and 15 |
| *CCP1*^pcd-3J^ | Spontaneous | Ataxia; degeneration of cerebellar Purkinje cells and granule cells; degeneration of thalamus neurons, olfactory bulb mitral cells; degeneration of photoreceptor cells; male infertility, female partial fertility; degeneration of sperm. Reduced mRNA of lower size and no detectable protein. | Deletion of exons 7, 8 and 9 |
| *CCP1*^pcd-4J^ | Chemically induced (ENU) | Ataxia; degeneration of cerebellar Purkinje cells. | Unknown |
| *CCP1*^pcd-5J^ | Spontaneous | Ataxia; degeneration of cerebellar Purkinje cells; degeneration of photoreceptor cells; male infertility. Normal mRNA levels but reduced protein levels due to instability. | Insertion of an aspartic acid codon in exon 18 |
| *CCP1*^pcd-6J^ | Chemically induced (ENU) | Ataxia; degeneration of cerebellar Purkinje cells, mitral cells in the olfactory bulb, the ventral root of the spinal cord; degeneration of retina; small muscle fibers; male infertility, degeneration of sperm, testicular atrophy. | Unknown |
| *CCP1*^pcd-7J^ | Spontaneous | Ataxia; degeneration of cerebellar Purkinje cells; enlarged hippocampus; abnormal hearing. | Unknown |
| *CCP1*^pcd- Tg(Dhfr)1Jwg^ | Transgenic (random, gene disruption) | Ataxia; degeneration of cerebellar Purkinje cells; degeneration of olfactory bulb mitral cells; degeneration of retinal photoreceptor cells; some male infertility, female partial fertility; degeneration of sperm. | Unknown |
| *CCP1*^babe^ | Chemically induced | ataxia；paraparesis | P804 arginine to a termination codon |
| *CCP1*^m2Btlr^ | Chemically induced | tremors；decreased body size；reduced activated sperm motility | an A to G transition; It destroys the acceptor splice site of intron 7 of the gene |
| *CCP1*^pcd-Btlr^ | Chemically induced | ataxia；nervous system phenotype；Purkinje cell degeneration；retinal photoreceptor degeneration；oligozoospermia；teratozoospermia；male infertility | a T-to-A transversion in the donor splice site of intron 11 |
| *CCP1*^Gt(IST13517F11)Tigm^ | Gene trapped allele | one ES cell; unclassified | Chr13:59477801-59478055 bp (-)；Chr13:59477801-59477979 bp (-) |
| *CCP1*^Gt(OST186151)Lex^ | Gene trapped allele | Lex-1 (ES Cell) | Chr13:59531904-59544452 bp (-) |

| Supplementary Table 1. continued - The summary of reported *CCP1* alleles | | | |
| --- | --- | --- | --- |
| *CCP1*^Gt(OST188387)Lex^ | Gene trapped allele | Lex-1 (ES Cell) | Chr13:59531902-59533237 bp (-) |
| *CCP1*^Gt(OST252171)Lex^ | Gene trapped allele | Lex-1 (ES Cell) | Chr13:59531904-59544452 bp (-) |
| *CCP1*^Gt(OST300426)Lex^ | Gene trapped allele | Lex-1 (ES Cell) | Chr13:59531904-59544452 bp (-) |
| *CCP1*^Gt(OST300428)Lex^ | Gene trapped allele | Lex-1 (ES Cell) | Chr13:59536248-59536374 bp (-) |
| *CCP1*^Gt(OST301743)Lex^ | Gene trapped allele | Lex-1 (ES Cell) | Chr13:59531913-59536374 bp (-) |
| *CCP1*^pcd-8J^ | Spontaneous allele | [ataxia；decreased body size；decreased Purkinje cell number；abnormal male germ cell morphology；reduced female fertility；male infertility；abnormal retinal layer morphology；retinal degeneration](http://www.informatics.jax.org/searches/Phat.cgi?id=MP:0001393) | allelic with Purkinje cell degeneration 3J |
| Note: Modified from Wang et al. 2007. | | | |
