# Supplementary material for: Lack of Cytosolic Carboxypeptidase 1 Leads to Subfertility due to the Reduced Number of Antral Follicles in pcd3J-/- Females
Source: PLoS One. 2015 Oct 9;10(10):e0139557. doi: 10.1371/journal.pone.0139557 (PMC4599934; doi:10.1371/journal.pone.0139557)
Supplement: S2 Table — (DOC) [file pone.0139557.s003.doc]

| **S2 Table.** Comparison of body weights between *pcd^3J+/+^* and *pcd^3J-/-^*female mice at 8 weeks old. | |
| --- | --- |
| Genotype | Body weight (g) |
| *pcd^3J+/+^* | 18.0±1.4 (n=5) |
| *pcd^3J-/-^* | 11.8±1.8 (n=3) |
| n, the number of mice used. | |
